# A synergistic drug combination that aids our battle against anaplastic thyroid carcinoma: a case report and discussion

**DOI:** 10.1007/s42000-025-00738-z

**Published:** 2025-11-25

**Authors:** Estefanía Chumbiauca, Isabela Valdés Calero, José M. López Picazo, Marta Moreno-Jiménez, Allan Argueta, María D. Lozano, Leonidas Duntas, Juan C. Galofré

**Affiliations:** 1 Department of Endocrinology, Clínica Universidad de Navarra, Pamplona, Spain; 2 Department of Medical Oncology, Clínica Universidad de Navarra, Pamplona, Spain; 3 Instituto de Investigación Sanitaria de Navarra (IdiSNA), Clínica Universidad de Navarra, Pamplona, Spain; 4 Department of Radiation Oncology , Clínica Universidad de Navarra, Pamplona, Spain; 5 Department of Pathology, Clínica Universidad de Navarra, Pamplona, Spain; 6 Evgenideion Hospital, Unit of Endocrinology, Diabetes and Metabolism, National and Kapodistrian University of Athens, Athens, Greece

**Keywords:** Anaplastic thyroid carcinoma, Immunotherapy, PD-L1 expression, Immune checkpoint inhibitors, Long-term survival

## Abstract

**Background:**

Anaplastic thyroid carcinoma (ATC) is a rare and highly aggressive malignancy historically associated with a poor prognosis and a median survival of only 3.16 months. However, recent advances in molecular profiling and immunotherapy are improving outcomes.

**Case presentation:**

We report the case of a 68-year-old man diagnosed with stage IVC ATC who achieved sustained disease stabilisation and long-term survival. Initial treatment with chemotherapy and radiotherapy led to partial remission of the primary tumour, although there was progression of metastatic disease. The patient was subsequently enrolled in a clinical trial investigating an anti-PD-L1 antibody with positive test results, followed by treatment with pembrolizumab. The latter resulted in nearly 11 years of disease control without significant adverse effects, an exceptional outcome in the context of ATC.

**Conclusion:**

This case underscores the transformative potential of immunotherapy in the management of advanced ATC and highlights the importance of continued research into combinatorial therapeutic strategies and predictive biomarkers to optimise treatment outcomes.

## Introduction

In 480 BCE Leonidas (540 − 480 BCE), king of Sparta, led an alliance of approximately 7,000 Greek warriors (including 300 Spartans) to the Battle of Thermopylae, at which they faced a vastly superior Persian army under Xerxes I, estimated at about 300,000 men [1]. Despite overwhelming odds, the Greek resistance, though ultimately overrun, is remembered as a strategic and symbolic victory. This heroic historical endeavour could be said to parallel the immense challenges faced in the modern fight against anaplastic thyroid carcinoma (ATC), a malignancy characterised by relentless progression and poor outcomes.

ATC ranks among the most aggressive solid tumours [2] with a median overall survival of just 3.16 months [3]. However, improved survival has been observed in recent years due to the advent of personalised molecular therapies and multidisciplinary management [4–6]. The 2021 American Thyroid Association (ATA) guidelines recommend molecular profiling at diagnosis to guide the use of targeted therapies [7]. For example, the combination of dabrafenib and trametinib is approved for patients with BRAF V600E-mutant ATC [8].

Immunotherapy has emerged as another promising therapeutic avenue supported by studies demonstrating PD-L1 expression in ATC tumours [9, 10]. The ATA guidelines suggest considering PD-L1 inhibitors as first-line treatment in the absence of other actionable mutations or as a final-line option [7].

We present a case of stage IVC ATC with a favourable response to a multimodal therapeutic approach, including anti-PD-1 immunotherapy with pembrolizumab. This case is notable for the patient’s sustained disease stabilisation and long-term survival, an uncommon outcome in this clinical context.

## Case presentation

A 68-year-old man presented to our institution in July 2014 for a second opinion regarding a rapidly enlarging, firm cervical mass. His past medical history was unremarkable aside from chronic tobacco use since age 35 and a 2 cm thyroid nodule identified in 2005 that had not been further investigated.

On physical examination, a 5 cm firm mass was palpated in the right thyroid lobe. Cervical CT imaging confirmed a 5.7 cm right thyroid nodule with tracheal and oesophageal invasion, along with paratracheal lymphadenopathy (largest node: 1 cm). Ultrasound revealed a 5.5 cm solid hypoechoic lesion occupying most of the right lobe and infracentimetric right lateral cervical lymphadenopathy.

**Fig. 1:**
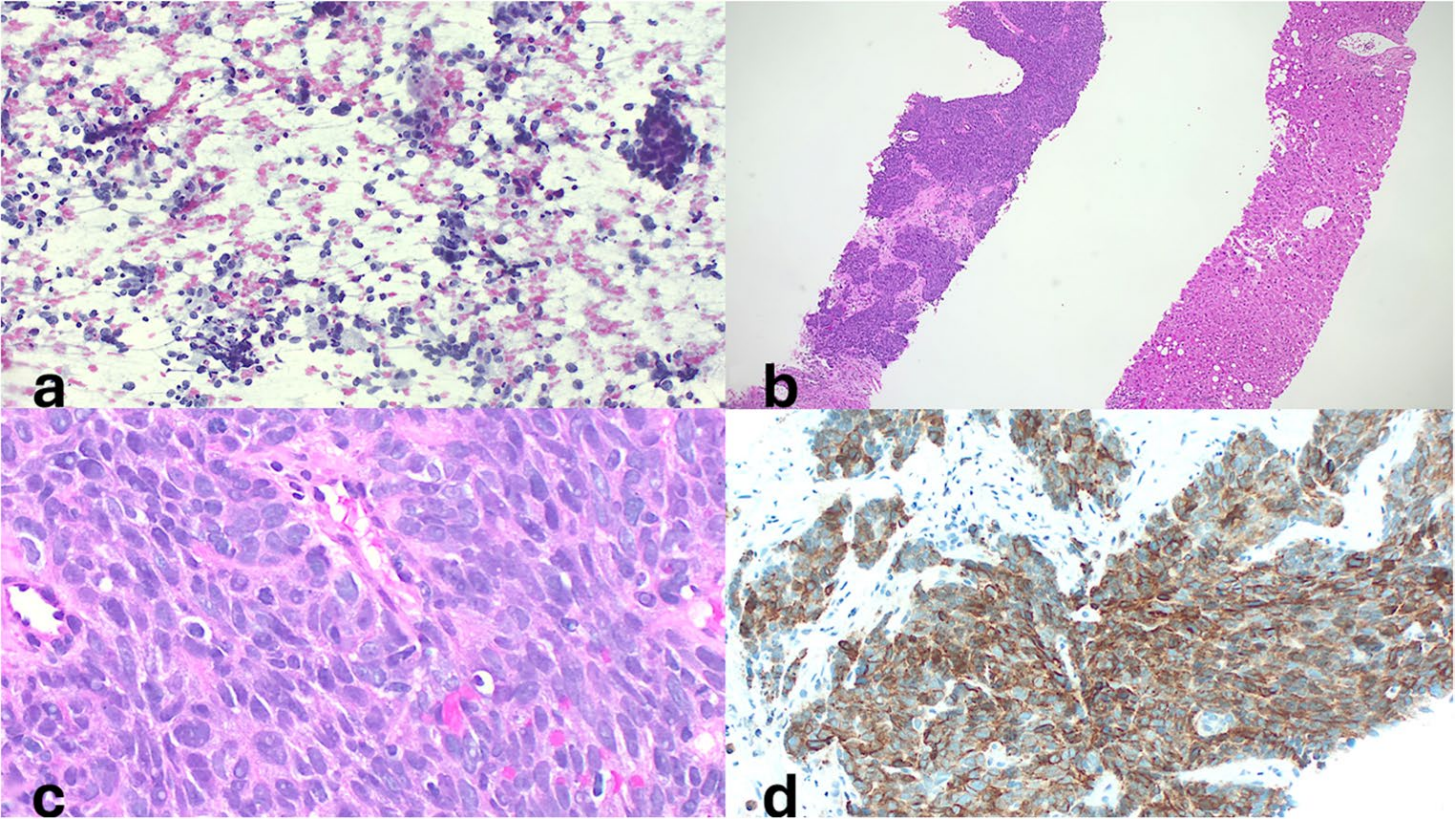
Histopathological Findings. **a**) Fine-needle aspiration of a thyroid nodule shows a cytological smear with moderate cellularity, composed of oval to elongated cells of medium to large size, exhibiting occasional intranuclear grooves, nucleoli, and scant cytoplasm. The cells are arranged either in sheets or as single units. A dirty background and a mild inflammatory infiltrate composed of lymphocytes are also observed. **b**–**c**) Liver biopsy obtained via ultrasound-guided percutaneous technique demonstrates metastatic involvement. **d**) Immunohistochemical staining for PD-L1 (SP263, Ventana Roche) performed on the liver biopsy reveals a Tumour Proportion Score (TPS) of 90%

Fine-needle aspiration biopsy indicated poorly differentiated thyroid carcinoma with features consistent with anaplastic carcinoma (Fig. 1a), likely arising from prior papillary thyroid carcinoma. Immunohistochemistry was positive for TTF-1 and negative for thyroglobulin and calcitonin. BRAF mutation testing was negative; TP53 and TERT mutations were not assessed. At that time (2014), comprehensive molecular profiling using next-generation sequencing (NGS) panels, including gene fusion analysis, was not routinely available in clinical practice.

**Fig. 2:**
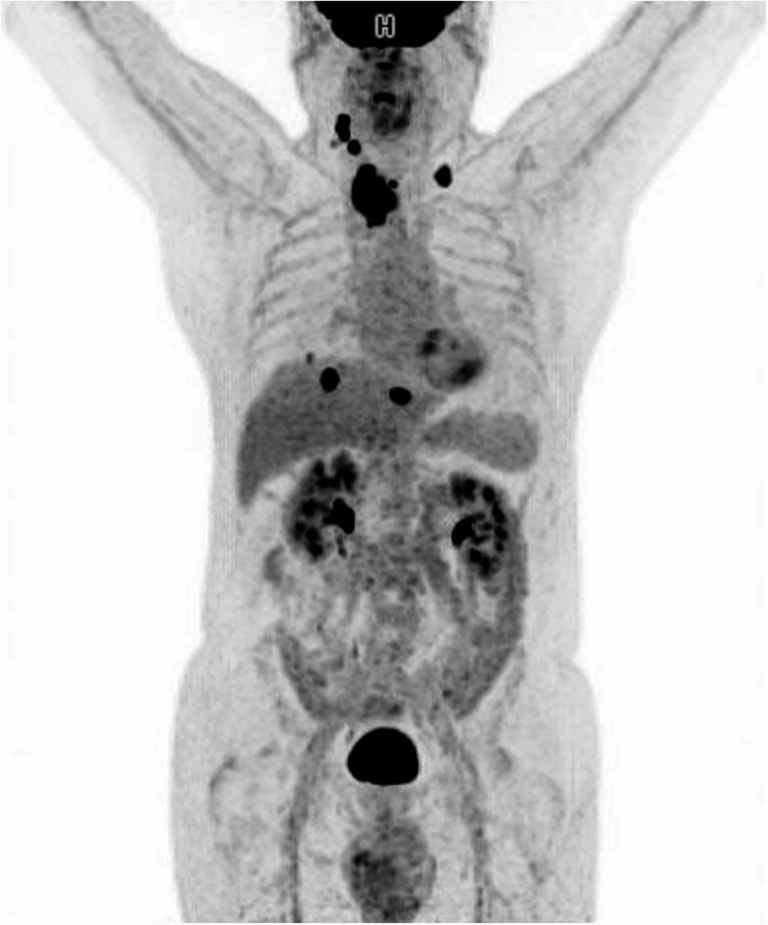
Initial 18F-FDG PET-CT. Positron emission tomography with 18F-fluorodeoxyglucose (18F-FDG PET/CT) at diagnosis revealed a primary lesion in the right thyroid lobe with tracheal extension (SUVmax: 9.30), along with bilateral lymph node involvement: right lateral cervical nodes (levels IIA and IIB, SUVmax: 4.53) and left retroclavicular node (SUVmax: 4.03). Additionally, multiple hypermetabolic hepatic foci suggestive of metastatic spread were identified, including one intrahepatic lesion in segment VIII (SUVmax: 6.24) and two peripheral lesions in segments II and VIII (SUVmax: 4.84 and 2.30, respectively)

Staging via 18 F-FDG PET-CT demonstrated a metabolically active primary thyroid tumour in the right lobe (SUVmax 9.3) with tracheal invasion, bilateral cervical lymphadenopathy (SUVmax 4.5 on the right and 4.0 on the left), and multiple hepatic metastases (SUVmax up to 6.2) (Fig. 2).

Initial treatment with cyclophosphamide, adriamycin, and cisplatin began in July 2014, yielding partial remission of the primary tumour. In September–October 2014, external beam radiotherapy (64 Gy) was delivered to the cervical region simultaneously with a single cycle of chemotherapy. Treatment was halted due to grade III oesophagitis. An 18-FDG PET-CT in February 2015 showed complete metabolic response in the thyroid, but revealed progression of hepatic metastases (SUVmax up to 9.9) and the appearance of a new bone lesion in D11 (SUVmax 1.7), consistent with metabolic progression involvement. Subsequent interventions included yttrium-90 radioembolisation for liver metastases (1 GBq administered intra-arterially to segment VIII; estimated absorbed doses: 286 Gy to tumour, 211 Gy to healthy liver, 2 Gy to lung) and stereotactic body radiotherapy to a solitary vertebral lesion at D11 (24.37 Gy delivered to 95% of the GTV in three alternate-day fractions), with excellent tolerance and favourable response.

In July 2015, disease progression was noted in the liver and left hilar lymph nodes. In August 2015 the patient was enrolled in a phase II clinical trial evaluating atezolizumab, an anti-PD-L1 antibody. The disease remained stable until January 2017 when pulmonary progression prompted withdrawal. Thoracic CT revealed enlargement of several bilateral pulmonary nodules, new nodules, mediastinal and hilar lymphadenopathy, and increased right pleural effusion with pleural implants, consistent with disease progression according to the RECIST 1.1 criteria. No treatment-related toxicity was observed.

In February 2017, a liver biopsy confirmed metastatic undifferentiated thyroid carcinoma with extensive tumour necrosis and residual, poorly differentiated carcinoma cells strongly positive for CK5/6 and negative for TTF-1 and c-KIT (Fig. 1b–c). Immunohistochemical staining for PD-L1 (SP263, Ventana Roche) performed on the liver biopsy demonstrated a tumour proportion score of 90% (Fig. 1d). Pleural fluid cytology showed chronic lymphocytic inflammation without malignant cells. Pembrolizumab (off-label) was initiated.

The patient experienced sustained disease stabilisation under pembrolizumab. In February 2024, immunotherapy was discontinued following at least 2 years of complete response. Minor side effects—xerostomia and a transient morbilliform rash—resolved with symptomatic treatment.

As of July 2025, 17 months after pembrolizumab discontinuation, the patient remains in complete remission nearly 11 years post-diagnosis and in excellent general condition (ECOG 0).

The patient’s therapeutic course and clinical response are summarised chronologically in Table 1.

**Table 1:** Timeline of treatment and response

Date	Treatment	Outcome
Jul 2014	Two cycles chemotherapy (cyclophosphamide, adriamycin, and cisplatin)	Partial remission of the primary tumour; findings consistent with baseline disease (RECIST 1.1)
Sep-Oct 2014	External beam radiotherapy (64 Gy) to the cervical region + one cycle chemotherapy	Chemotherapy suspended due to oesophagitis grade III during radiotherapy
Feb 2015	18-FDG PET-CT	Complete local metabolic response in the thyroid Progression of hepatic metastases (SUVmax up to 9.9) and possible bone metastasis in D11 (SUVmax 1.7), consistent with progressive disease (RECIST 1.1)
Feb-Apr 2015	Yttrium-90 radioembolisation for hepatic metastases (1 GBq, segment VIII; estimated absorbed dose: tumour 286 Gy, healthy liver 211 Gy, lung 2 Gy); stereotactic body radiotherapy to D11 metastasis (24.37 Gy in 3 fractions)	Good response and excellent tolerance
Jul 2015	18-FDG PET-CT	Liver and left hilar lymph node recurrence, consistent with progressive disease (RECIST 1.1)
Aug 2015-Jan 2017	Atezolizumab clinical trial	Pulmonary progression (RECIST 1.1) led to withdrawal from trial. No treatment-related toxicity
Feb 2017-Feb 2024	Pembrolizumab	Sustained disease stabilisation led to complete response (RECIST 1.1) after ≥ 2 years of therapy. No treatment-related toxicity
Mar 2024-July 2025	Clinical follow-up. Pembrolizumab withdrawal	Complete response maintained, patient in excellent general condition (ECOG 0)

## Discussion

ATC is associated with an exceptionally high mortality rate and timely diagnosis is critical due to its rapid progression [7]. In most cases, including ours, the disease is disseminated at presentation. Approximately 20–50% of ATC cases arise from pre-existing differentiated thyroid carcinoma [11].

Historically, treatment has included surgery (when feasible), chemotherapy, and radiotherapy [8].

According to the 2021 ATA guidelines, surgery is recommended for stage IVA–IVB disease when complete (R0/R1) resection is possible, but generally not for stage IVC due to involvement, by definition, of distant metastases [7]. In disseminated disease, systemic therapy and radiotherapy are emphasised, with surgery reserved for palliation or control of life-threatening complications. Nonetheless, retrospective studies and systematic reviews have reported improved survival in some patients undergoing surgery despite metastases [12], though this is likely influenced by selection bias. In a systematic review, surgery conferred a survival benefit in patients with stage IVB but not with IVC, and negative margins (R0) showed no clear advantage over R1/R2 resections [13]. Contemporary series suggest that in highly selected stage IVC patients, particularly those responding favourably to systemic or targeted therapy, surgery may be considered for local control or symptom relief, employing a multimodal strategy, although the evidence is retrospective and remains limited.

In our case, surgery was not feasible and the patient received systemic therapy instead. Despite initial positive of the primary tumour, distant metastases progressed, highlighting the limited efficacy of traditional therapies and the need for novel therapeutic strategies.

Modern management increasingly incorporates genetic and molecular profiling to guide personalised therapy [7, 14]. In tumours harbouring the BRAF V600E mutation (25–45% of ATCs), BRAF/MEK inhibition (dabrafenib plus trametinib) achieves high response rates and survival benefits and is now recommended as first-line therapy [7, 8]. Furthermore, this regimen received tumour-agnostic FDA accelerated approval in 2022 for unresectable or metastatic solid tumours with a BRAF V600E mutation, supporting its use as a precision therapy [15]. Unfortunately, our patient was BRAF-negative, precluding this strategy.

Molecular testing may be further expanded by the search for other actionable genetic alterations, such as NTRK, RET, ALK, or ROS1 fusions, for which targeted therapies are available (e.g., larotrectinib, entrectinib, and selpercatinib) [14]. Larotrectinib and entrectinib are tumour-agnostic therapies for NTRK fusions, while selpercatinib is approved for RET fusion-positive thyroid cancers. Although ALK and ROS1 fusions are rare in ATC, NGS can identify them and thereby enable access to matched therapies in selected cases.

Additionally, immune checkpoint inhibitors (ICIs) provide a valuable option for selected patients. The PD-1/PD-L1 interaction inhibits antitumour immune activity, allowing tumour immune evasion [14, 16]. Monoclonal antibodies blocking this pathway have demonstrated clinical benefit across multiple cancers [17]. PD-L1 is expressed in up to 65–90% of ATCs [14, 18]. Preclinical studies show that combining PD-L1 inhibitors with targeted agents (e.g., BRAF/MEK inhibitors) enhances tumour suppression [19].

Clinical experience with ICIs in thyroid malignancies is growing: phase Ib and phase II trials in PD-L1–positive differentiated thyroid cancer and advanced thyroid cancer, respectively, showed favourable safety and durable responses in a subset of patients [20, 21].

Some strategies seek to improve efficacy through synergistic combinations. In BRAF V600E-mutated ATC, combining ICIs with targeted therapy has shown encouraging outcomes [9], supporting the integration of immune checkpoint blockade —either alone or in rational combinations—into the therapeutic landscape of ATC. Notably, lenvatinib plus pembrolizumab has demonstrated promising activity in both ATC and poorly differentiated thyroid carcinoma [20]. Such combinations may be particularly relevant for patients lacking actionable genomic alterations. Consequently, routine NGS upon ATC diagnosis is strongly recommended whenever feasible as it enables the identification of clinically actionable alterations that can guide therapy and improve outcomes [7, 14].

In our patient, comprehensive molecular profiling was not available at diagnosis and testing for PD-L1 expression had not been performed prior to enrolment in the atezolizumab phase II trial, as it was not yet standard practice. After more than 1 year of stable disease, pulmonary progression in early 2017 led to the discontinuation of atezolizumab. A subsequent liver metastasis biopsy revealed high PD-L1 expression (TPS 90%), supporting the initiation of off-label pembrolizumab, which achieved a durable complete response. This exceptional outcome highlights both the prognostic and predictive value of PD-L1 and underscores the importance of individualised immune-based therapeutic strategies.

## Conclusions

ATC is a highly aggressive malignancy with a historically grim prognosis. However, advances in molecular profiling and immunotherapy are reshaping clinical outcomes. This case demonstrates the potential of pembrolizumab to achieve long-term disease control with minimal toxicity. Ongoing research into combinatorial strategies and biomarker identification is critical to further improve treatment outcomes.
